# An Important Role of Blood and Lymphatic Vessels in Inflammation and Allergy

**DOI:** 10.1155/2013/672381

**Published:** 2013-01-31

**Authors:** Silvana Zgraggen, Alexandra M. Ochsenbein, Michael Detmar

**Affiliations:** Institute of Pharmaceutical Sciences, Swiss Federal Institute of Technology, ETH Zurich, Wolfgang Pauli-Strasse 10, HCI H303, 8093 Zurich, Switzerland

## Abstract

Angiogenesis and lymphangiogenesis, the growth of new vessels from preexisting ones, have received increasing interest due to their role in tumor growth and metastatic spread. However, vascular remodeling, associated with vascular hyperpermeability, is also a key feature of many chronic inflammatory diseases including asthma, atopic dermatitis, psoriasis, and rheumatoid arthritis. The major drivers of angiogenesis and lymphangiogenesis are vascular endothelial growth factor- (VEGF-)A and VEGF-C, activating specific VEGF receptors on the lymphatic and blood vascular endothelium. Recent experimental studies found potent anti-inflammatory responses after targeted inhibition of activated blood vessels in models of chronic inflammatory diseases. Importantly, our recent results indicate that specific activation of lymphatic vessels reduces both acute and chronic skin inflammation. Thus, antiangiogenic and prolymphangiogenic therapies might represent a new approach to treat chronic inflammatory disorders, including those due to chronic allergic inflammation.

## 1. Introduction

According to the World Allergy Organization, allergic disorders affect 30–40% of the world's population, and the prevalence is escalating to epidemic proportions. Much of the pathology of chronic allergic disorders such as atopic dermatitis and asthma is the long-term result of chronic allergic inflammation at the site of allergen exposure [1]. Thus, to explore additional possibilities to treat chronic allergic disorders, it is of importance to understand the distinct pathomechanisms and properties of chronic inflammation. 

Inflammation in general is the response of tissues to harmful stimuli such as infectious agents, antigens, or physical and chemical damage. Besides the increased inflammatory cell infiltration into the inflamed tissue, it has become clear in the recent years that acute and chronic inflammatory processes are associated with pronounced vascular remodeling. Angiogenesis and lymphangiogenesis, the growth of new blood vessels and of lymphatic vessels from preexisting ones, are involved in a number of physiological and pathological conditions such as wound healing, tumor growth, and metastatic spread [2–5]. Angiogenesis and lymphangiogenesis also occur in several chronic inflammatory conditions, including rheumatoid arthritis, inflammatory bowel disease, asthma, chronic airway inflammation, atopic dermatitis, and psoriasis [6–9]. 

Even though blood and lymphatic vessels are key players in acute and chronic inflammatory processes, and thus might serve as new therapeutic targets in inflammatory and allergic diseases, there is currently no clinically approved treatment to specifically modulate the vasculature. 

## 2. The Function of Blood Vessels and Lymphatic Vessels in Tissue Homeostasis

In vertebrates, there are two vascular systems: the cardiovascular and the lymphatic system. To exert their functions, both vascular systems build highly branched, tree-like tubular structures. In the cardiovascular system, the heart pumps the blood through arteries into smaller arterioles and into capillary beds. From there, the blood returns via venules and veins to the heart to proceed to the lungs for new oxygen loading. Under physiological conditions, the major functions of blood vessels include the supply of gases, fluid, nutrition, and signaling molecules to the tissues with the capillaries as the actual sites of exchange. At these sites, plasma leaks from the capillaries into the interstitium, driven by blood pressure and osmotic gradients. The lymphatic capillaries take up this protein-rich fluid, thereby maintaining not only tissue fluid homeostasis but also exerting immune surveillance. The lymphatic network is composed of blind-beginning thin-walled capillaries without pericyte coverage and with incomplete basal lamina as well as of collecting lymphatic vessels with a smooth muscle cell layer, a basement membrane, and valves, which prevent back flow of lymph. The largest collecting lymphatic vessel, the thoracic duct, connects the lymphatic system with the cardiovascular system. In adults, physiological angiogenesis and lymphangiogenesis are uncommon. However, new lymphatic and blood vessels form during the female reproductive cycle, the hair cycle, and in healing wounds [10, 11]. 

## 3. Anatomy of the Cutaneous and Pulmonary Vascular Systems

The epidermal layer of the skin is free of blood and lymphatic vessels. In the dermis, the blood vascular system is organized into a deep and a superficial horizontal plexus with capillaries arising from the latter one [12, 13]. The lymphatic vasculature also forms two plexuses in vicinity to the blood vessels. Branches from the superficial lymphatic vessel plexus extend into the dermal papillae and descend into the larger lymphatic vessels in the lower dermis [14]. The bulk of blood microvascular vessels is located immediately below the epidermis, whereas the lymphatic vessels reside more distant to the epidermis [15]. 

Pulmonary blood vessels transport the low-oxygen blood from the heart to the lung, whereas the bronchial vessels supply the lung with nutrients and oxygen. The bronchial blood vessels arise from the aorta or intercostal arteries and enter the lung at the hilum. At the main stem bronchus, they branch and descend to the lower trachea and extrapulmonary airways. They cover the whole lung from the bronchial tree to the terminal bronchioles, where the bronchial vessels anastomose with each other as well as with pulmonary vessels [16]. Studies of lymphatic vessels in normal human lung are rare, not only because for a long time lymphatic specific markers were not known, but also because in the lung the commonly used lymphatic marker lymphatic vessel endothelial hyaluronan receptor- (LYVE-)1 stains both lymphatic and blood vascular endothelial cells [17]. Recent studies using the specific lymphatic endothelium marker podoplanin showed that in human lung, lymphatic vessels extend beyond the respiratory bronchioles, accompanying intralobular arteries deep inside the lobule, the smallest unit of the lung [18, 19]. In the murine trachea, the lymphatic network is highly ordered, and vessels are restricted to the mucosa located between the cartilage rings [8]. 

## 4. The Role of Blood and Lymphatic Vessels in Inflammation

Both the blood and the lymphatic vascular system contribute to the body's inflammatory response. In acute inflammation, blood vascular endothelial cells are activated by several inflammatory mediators (e.g., vascular endothelial growth factor- (VEGF-)A, tumor necrosis factor (TNF-)*α*, interleukin (IL)-6, and IL-1*β*), leading to the typical signs of inflammation, increased blood flow as a consequence of vessel dilation and edema formation due to increased permeability of the blood vessels. Furthermore, the expression of adhesion molecules, such as intercellular adhesion molecule- (ICAM-)1, vascular cell adhesion molecule- (VCAM-)1, and E-selectin on activated blood vascular endothelial cells, enables the interaction between leukocytes and endothelium, a major event in the inflammatory process [20, 21]. In chronic inflammation, the blood vasculature remains enlarged, hyperpermeable, and activated with high expression of adhesion molecules, leading to continuous extravasation of inflammatory cells and fluid into the inflamed tissue. A number of inflammatory conditions such as rheumatoid arthritis, inflammatory bowel disease, asthma, atopic dermatitis, and psoriasis are characterized by pronounced angiogenesis [7, 9, 22, 23]. 

Besides the blood vasculature, also the lymphatic vasculature plays an important role in inflammation. Lymphatic vessels regulate the inflammatory response by the transport of fluid, extravasated leukocytes, and antigen-presenting cells from the inflamed tissue to the lymph nodes and to other secondary lymphoid organs, thereby contributing to the decrease of inflammation-induced edema and to the initiation of a specific immune response. The C-C chemokine receptor type (CCR) 7 expressed by dendritic cells is important for their migration into afferent lymphatic vessels which secrete the respective ligand chemokine (C-C motif) ligand (CCL) 21 [24]. Lymphangiogenesis occurs in several chronic inflammatory conditions such as human psoriasis and mouse models of chronic skin inflammation, chronic airway inflammation, and rheumatoid arthritis [8, 25–27]. 

## 5. Mediators of Angiogenesis and Lymphangiogenesis in Inflammation

**Figure 1:**
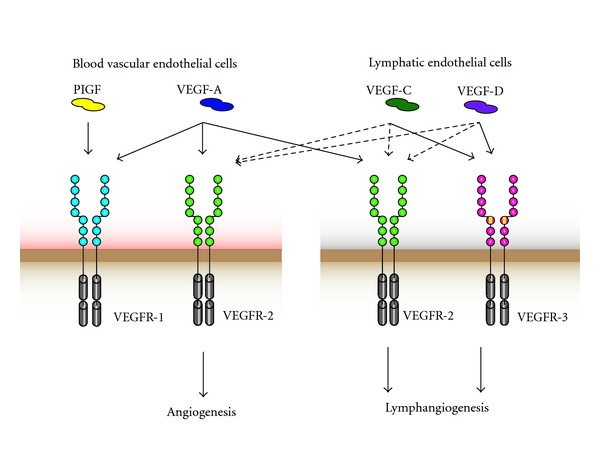
VEGF-binding properties and distinct VEGF receptor expression on lymphatic and blood vascular endothelium. VEGFs bind to the three VEGF receptor tyrosine kinases, leading to the formation of VEGFR dimers. Blood vascular endothelial cells express VEGFR-1 and VEGFR-2, whereas lymphatic endothelial cells express VEGFR-2 and VEGFR-3. VEGF-A—which binds both VEGFR-1 and VEGFR-2—can directly induce blood and lymphatic vascular remodeling. VEGF-C and -D bind VEGFR-3 and, after proteolytic processing, also VEGFR-2, thus inducing angiogenesis and lymphangiogenesis.

In recent years, the understanding of inflammatory angiogenic and lymphangiogenic processes such as endothelial cell growth, migration, and survival has increased, and a variety of involved mediators have been identified. The most important molecule that controls inflammation-driven angiogenesis is VEGF-A, a member of a family of angiogenic and lymphangiogenic drivers such as VEGF-C, VEGF-D, and placenta growth factor (PlGF) [6, 28]. VEGF-A signals via its receptor tyrosine kinases VEGFR-1 and VEGFR-2 and thereby induces angiogenesis and lymphangiogenesis (Figure 1). Expression of VEGF-A and VEGFR-2 is induced by cytokines such as TNF-*α*, thereby linking angiogenesis with inflammatory conditions [14, 29]. VEGFR-1 is expressed on blood vessels, whereas VEGFR-2 is expressed on both blood and lymphatic vessels with high expression on tip cells [30]. Compared to VEGFR-2, VEGFR-1 has a higher affinity for VEGF-A but a lower kinase activity. VEGFR-1 has been reported to trap VEGF-A to prevent excess signaling via VEGFR-2 during embryogenesis, whereas in the adult the function of VEGFR-1 remains more elusive [31, 32]. Several cytokines and chemokines such as IL-1, IL-8, IL-18, chemokine (C-X-C motif) ligand (CXCL) 3, and CXCL12 have also been reported to exert proangiogenic and lymphangiogenic activities (Table 1). 

Inflammatory lymphangiogenesis, as lymphangiogenesis in general, is mainly driven by VEGFR-2 and -3 signaling. Thus, VEGF-A-induced VEGFR-2 signaling plays not only a role in angiogenesis but also in lymphangiogenesis. VEGFR-3, which is expressed on lymphatic endothelial cells, binds VEGF-C and VEGF-D (Figure 1). However, after proteolytic cleavage, VEGF-C can also induce VEGFR-2 signaling [33–35]. VEGFR-2 and -3 signaling have been shown to be involved in inflammatory lymphangiogenesis in mouse models of skin inflammation and of airway inflammation [8, 36, 37]. These models will be discussed in more detail later on. The expression of the lymphangiogenic factor VEGF-C is induced in response to different proinflammatory cytokines such as TNF-*α* and IL-1*β*, most likely via the activation of the nuclear factor of kappa light polypeptide gene enhancer in B-cells (NF-*κ*B) pathway [38, 39]. Furthermore, inflammatory cells such as macrophages promote the formation of lymphatic vessels by secreting VEGF-C and VEGF-D [8, 40]. TNF-*α* might also contribute to inflammatory airway lymphangiogenesis [39]. 

## 6. Blood and Lymphatic Vessels in Chronic Skin Inflammation

**Figure 2:**
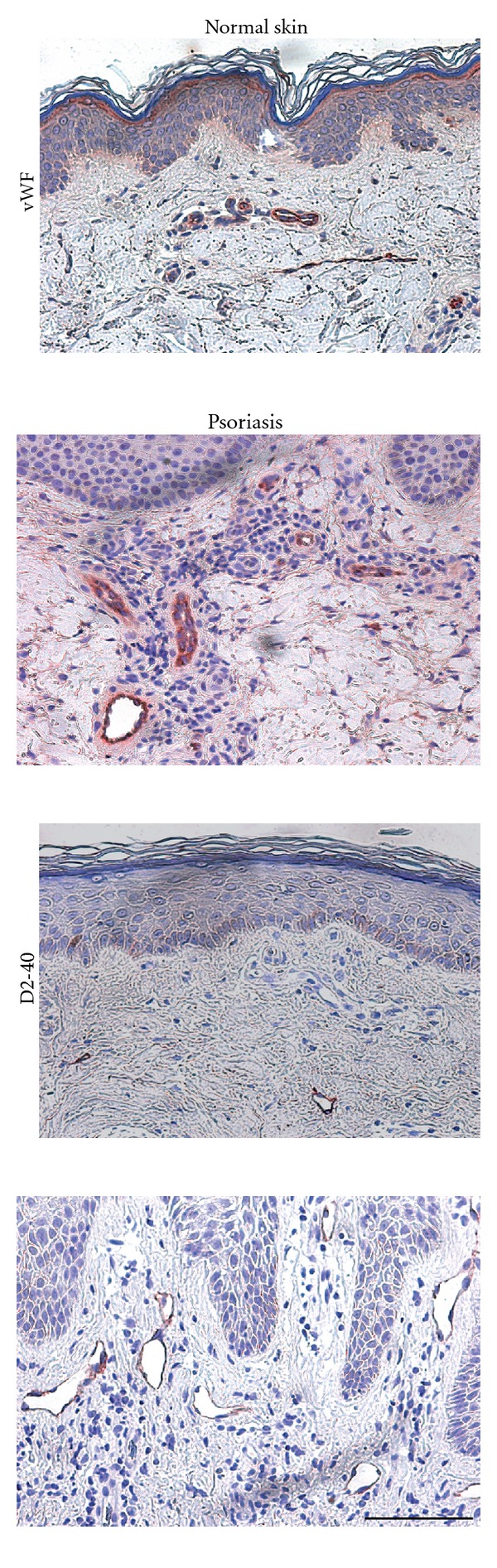
Blood and lymphatic vessel enlargement in human psoriasis. The number and size of von Willebrand factor (vWF)-positive blood vessels in lesional psoriatic skin are increased compared to normal healthy skin. Also the size of D2-40 positive lymphatic vessels is increased in lesional psoriatic skin. Bar = 100 *μ*m.

Several skin diseases such as atopic dermatitis, contact dermatitis, UV damage, and psoriasis are associated with increased vascular remodeling (Figure 2) [25, 41, 42]. In the lesional skin of atopic dermatitis and psoriasis, levels of the angiogenic growth factor VEGF-A are elevated [27, 43, 44], and in psoriasis patients, the plasma levels of VEGF-A correlate positively with the disease severity [45]. 

**Figure 3:**
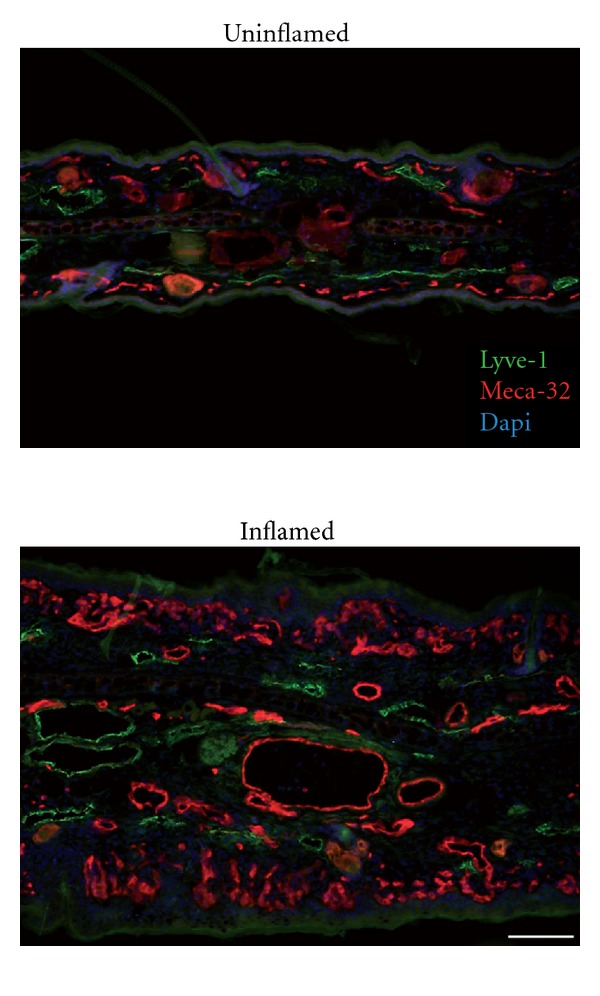
Blood and lymphatic vessel enlargement in the inflamed skin of K14-VEGF-A transgenic mice. The number and size of Meca-32 positive blood vessels (red) and LYVE-1 positive lymphatic vessels (green) are increased in the inflamed skin of K14-VEGF-A transgenic mice compared to uninflamed skin. Bar = 100 *μ*m.

In recent years, a number of mouse models have been developed to study vascular remodeling in chronic skin inflammation, for example, the epidermal specific JunB/C-Jun knockout mice [46], human psoriatic skin transplantation onto severe combined immunodeficiency- (SCID-)mice [47], and K14 (keratin14) VEGF-A transgenic mice [48] which have been developed in our laboratory. In these mice, murine VEGF-A164 is continuously expressed in epidermal keratinocytes under the control of the K14 promoter. Mice homozygous for this transgene develop a chronic cutaneous inflammation at the age of approximately 5-6 months, which has most of the features of human psoriasis, namely epidermal hyperplasia and abnormal terminal differentiation of epidermal keratinocytes, typical leukocyte infiltration including dermal CD4+ T cell and epidermal CD8+ T-cell accumulation as well as a pronounced increase in the number and size of blood and lymphatic vessels (Figure 3) [48]. In hemizygous K14-VEGF-A transgenic mice, the chronic skin inflammation is inducible by applying the contact sensitizer oxazolone [25]. Several studies in these K14-VEGF-A transgenic mice by our laboratory and others have validated these mice as a valuable model for chronic cutaneous inflammation with a relevant involvement of vascular remodeling. In this model, the small molecular VEGFR inhibitor NVP-BAW2881 showed strong anti-inflammatory actions with a reduction in inflammation-induced angiogenesis and lymphangiogenesis [49]. Furthermore, treatment with the specific anti-VEGFR-2 antibody DC101 inhibited skin inflammation, inflammatory cell infiltration, and angiogenesis [37], indicating that angiogenesis plays an important role for disease maintenance and progression. The importance of the lymphatic vasculature in inflammation was also studied in these mice. Surprisingly, blockade of VEGFR-3 signaling increased the severity of skin inflammation. Conversely, specific activation of the lymphatic vasculature by intracutaneous injections of recombinant VEGF-C156S, a specific ligand of VEGFR-3, reduced chronic skin inflammation [37]. Taken together, hemizygous K14-VEGF-A tg mice represent a reliable model to study inflammation-induced vascular remodeling in the skin and to test the potential effectiveness of new anti-inflammatory drugs. However, psoriasis is a human-specific disease that is not naturally observed in animals. Thus, different genetic and xenotransplant mouse models have been developed to mimic some features of human psoriasis [50]. Considering the substantial differences between mouse and human skin, however, most of these models do not fully recapitulate all characteristics of the human disease. Therefore, for specific pathogenetic studies, the evaluation of different psoriasis mouse models might be advantageous. In this regard, it has been recently reported that topical treatment with Imiquimod, a toll-like receptor 7 and 8 agonist, triggers psoriasis-like skin lesions in humans and in mice, with an involvement of the IL23/IL17 axis, known to play a crucial role in human psoriasis [51]. 

## 7. Blood Vessels in Asthma

Asthma is a chronic inflammatory disease of the airways that is characterized by airway hyperresponsiveness, episodic airflow limitations, and a decline in lung function. These symptoms are caused by chronic inflammation and airway remodeling, including increased thickness of the lamina reticularis [52, 53], smooth muscle hyperplasia/hypertrophy, and increased vascularity [54, 55] in small and large airways [56]. In the 1960s, Dunill demonstrated for the first time the involvement of blood vessels in asthma by showing swollen bronchial mucosa with dilated and congested capillaries in lung samples of subjects who died of acute asthmatic attacks [57]. Since then, increased airway vascularity was not only found in severe but also in mild asthmatic cases, and it is nowadays a well established finding that the number of blood vessels and the tissue area covered by blood vessels are increased in asthmatic patients compared to healthy subjects (reviewed in [55]). In mild asthma cases, the increased submucosal vascularity in the inner area of the medium airway might contribute to airflow limitation, as indicated by the inverse correlation of vascularity and forced expiratory volume, a measure for airway obstruction [56]. VEGF-A, the major driver of angiogenesis, was also increased in sputum samples of patients with mild asthma compared to healthy controls [58]. VEGF-A and VEGFR-1 mRNA levels were increased in lung biopsy specimens from patients with mild-to-moderate asthma compared to healthy controls. The reported colocalization of VEGF-A with CD68, major basic protein and Chymase-positive cells suggests that macrophages, eosinophils, and mast cells are a major source of VEGF-A in the lung [59, 60], which thus may contribute to angiogenesis in asthma. Conversely, it has been shown—in different *in vitro* and *in vivo* settings—that eosinophils, mast cells, and macrophages are also influenced by angiogenic factors. Due to their expression of VEGFR-1, these cells are able to migrate towards VEGF-A gradients [61–65]. Therefore, increased VEGF-A levels at the site of inflammation contribute to the recruitment of different inflammatory cells which themselves can secrete proinflammatory mediators. 

McDonald and colleagues established the *Mycoplasma pulmonis* infection model of chronic airway inflammation as a valuable tool for investigation of chronic inflammatory airway diseases in mice. This model shows several—though not all—characteristic features of asthma such as inflammatory cell influx, angiogenesis, mucosal edema, epithelial changes, fibrosis, and bronchial hyperreactivity [8, 66]. Shortly after *M. pulmonis* infection, mucosal blood vessels enlarge by endothelial cell proliferation, and angiogenesis reaches a plateau at 14 days after infection [8]. Surprisingly, VEGF receptor blocking studies showed that this pathological angiogenesis might not be driven by VEGF-A [8]. However, blocking of TNF-*α* signaling by an anti-TNF-*α* antibody dramatically reduced blood vessel remodeling 14 days after *M. pulmonis* infection, suggesting that TNF-*α* signaling is involved in this angiogenic process [39]. 

## 8. Lymphatic Vessels in Chronic Airway Disease

Edema formation results when the amount of leakage from the blood vessels exceeds the capacity of lymphatic vessels for drainage. Such edemas are a cardinal sign of chronic inflammation, and indeed increased microvascular permeability as well as edema are features of asthma [67]. However, knowledge of lymphatic involvement in edema formation in asthma is remarkably sparse. Only recently, it was shown that lymphangiogenesis increased with advanced stages of idiopathic pulmonary fibrosis, a chronic lung disease of unknown etiology with an insidious onset, leading to ventilatory restriction and respiratory failure [68]. 

In the *M. pulmonis* driven mouse model of chronic airway inflammation, there is also a dramatic remodeling of lymphatic vessels [8]. Robust lymphangiogenesis is most abundant on the surface facing the cartilage rings that grow towards the overlaying airway epithelium. Interestingly, after 4 weeks of treatment with antibiotics, lymphatic sprouting was completely inhibited, but lymphatic vessels regressed only partially, compared to the almost complete regression of blood vessels [8]. VEGF-C, VEGF-D, and TNF-*α* are major drivers of this remodeling. Blockade of VEGFR-3 signaling via soluble VEGFR-3-Fc or by an anti-VEGFR-3 antibody almost completely prevented lymphangiogenesis in tracheas of infected mice [8]. In contrast, adenoviral overexpression of VEGF-C in the murine trachea leads to enhanced lymphatic filopodia formation and to sprouts similar to those seen in the *M. pulmonis* infected mice. Immunofluorescence stainings showed that mucosal inflammatory cells, in particular F4/80+ macrophages, are a major source of VEGF-C in this model. Reduced lymphangiogenesis after infection with *M. pulmonis* was also observed after inhibition of TNF signaling by a blocking antibody and in TNFR-1 ko mice [39]. 

## 9. Conclusions and Outlook

There is clear evidence that in humans, vascular remodeling occurs in many chronic inflammatory disorders. Even though different anti-inflammatory drugs are on the market, there is no specific therapy that interferes with the pathological vascular changes that occur during inflammation. Angiogenesis and lymphangiogenesis are tightly linked to chronic inflammation, and targeting the blood vessels and lymphatic vessels has been shown to be an effective strategy in different experimental mouse models of chronic inflammation. One has to keep in mind, however, that in most conditions the vascular activation likely represents a downstream event that maintains the inflammatory process, but not the pathogenetic cause of the respective disease, which often has remained unknown. Nonetheless, antiangiogenic and prolymphangiogenic therapies might represent new approaches to treat chronic inflammatory disorders, including those due to chronic allergic inflammation. 

## Figures and Tables

**Table 1:** Proangiogenic cytokines and chemokines.

Name	References
Cytokines	
TNF-*α* ^+^	[69–71]
IL-1^+^	[72–74]
IL-6	[41]
IL-8 (CXCL18)	[75, 76]
IL-15	[77]
IL-17	[78]
IL-18^+^	[79–82]
Chemokines	
CXCL1	[83, 84]
CXCL2	[83, 84]
CXCL3	[83–86]
CXCL5	[83, 84]
CXCL6	[83, 84]
CXCL7	[83, 84]
CXCL9	[83]
CXCL12 (SDF-1)	[87, 88]
CCL2 (MCP-1)	[89, 90]

^+^Also antiangiogenic activity has been reported.

## References

[1] Galli SJ, Tsai M, Piliponsky AM (2008). The development of allergic inflammation. *Nature*.

[2] Carmeliet P (2003). Angiogenesis in health and disease. *Nature Medicine*.

[3] Karpanen T, Alitalo K (2008). Molecular biology and pathology of lymphangiogenesis. *Annual Review of Pathology*.

[4] Hirakawa S, Kodama S, Kunstfeld R, Kajiya K, Brown LF, Detmar M (2005). VEGF-A induces tumor and sentinel lymph node lymphangiogenesis and promotes lymphatic metastasis. *Journal of Experimental Medicine*.

[5] Mumprecht V, Detmar M (2009). Lymphangiogenesis and cancer metastasis. *Journal of Cellular and Molecular Medicine*.

[6] Detmar M, Brown LF, Claffey KP (1994). Overexpression of vascular permeability factor/vascular endothelial growth factor and its receptors in psoriasis. *Journal of Experimental Medicine*.

[7] Danese S, Sans M, de la Motte C (2006). Angiogenesis as a novel component of inflammatory bowel disease pathogenesis. *Gastroenterology*.

[8] Baluk P, Tammela T, Ator E (2005). Pathogenesis of persistent lymphatic vessel hyperplasia in chronic airway inflammation. *Journal of Clinical Investigation*.

[9] Thairu N, Kiriakidis S, Dawson P (2011). Angiogenesis as a therapeutic target in arthritis in 2011: learning the lessons of the colorectal cancer experience. *Angiogenesis*.

[10] Karkkainen MJ, Petrova TV (2000). Vascular endothelial growth factor receptors in the regulation of angiogenesis and lymphangiogenesis. *Oncogene*.

[11] Norrmén C, Tammela T, Petrova TV, Alitalo K (2011). Biological basis of therapeutic lymphangiogenesis. *Circulation*.

[12] Braverman IM (1989). Ultrastructure and organization of the cutaneous microvasculature in normal and pathologic states. *Journal of Investigative Dermatology*.

[13] Detmar M, Hirakawa S (2012). Vascular Biology. *Dermatology*.

[14] Huggenberger R, Detmar M (2011). The cutaneous vascular system in chronic skin inflammation. *Journal of Investigative Dermatology*.

[15] Skobe M, Detmar M (2000). Structure, function, and molecular control of the skin lymphatic system. *Journal of Investigative Dermatology Symposium Proceedings*.

[16] Zanini A, Chetta A, Imperatori AS, Spanevello A, Olivieri D (2010). The role of the bronchial microvasculature in the airway remodelling in asthma and COPD. *Respiratory Research*.

[17] Gordon EJ, Gale NW, Harvey NL (2008). Expression of the hyaluronan receptor LYVE-1 is not restricted to the lymphatic vasculature; LYVE-1 is also expressed on embryonic blood vessels. *Developmental Dynamics*.

[18] Kambouchner M, Bernaudin JF (2009). Intralobular pulmonary lymphatic distribution in normal human lung using D2-40 antipodoplanin immunostaining. *Journal of Histochemistry and Cytochemistry*.

[19] Sozio F, Rossi A, Weber E (2012). Morphometric analysis of intralobular, interlobular and pleural lymphatics in normal human lung. *Journal of Anatomy*.

[20] Pober JS, Sessa WC (2007). Evolving functions of endothelial cells in inflammation. *Nature Reviews Immunology*.

[21] Jackson JR, Seed MP, Kircher CH, Willoughby DA, Winkler JD (1997). The codependence of angiogenesis and chronic inflammation. *The FASEB Journal*.

[22] Ribatti D, Puxeddu I, Crivellato E, Nico B, Vacca A, Levi-Schaffer F (2009). Angiogenesis in asthma. *Clinical and Experimental Allergy*.

[23] Zhang Y, Matsuo H, Morita E (2006). Increased production of vascular endothelial growth factor in the lesions of atopic dermatitis. *Archives of Dermatological Research*.

[24] Ohl L, Mohaupt M, Czeloth N (2004). CCR7 governs skin dendritic cell migration under inflammatory and steady-state conditions. *Immunity*.

[25] Kunstfeld R, Hirakawa S, Hong YK (2004). Induction of cutaneous delayed-type hypersensitivity reactions in VEGF-A transgenic mice results in chronic skin inflammation associated with persistent lymphatic hyperplasia. *Blood*.

[26] Kajiya K, Detmar M (2006). An important role of lymphatic vessels in the control of UVB-induced edema formation and inflammation. *Journal of Investigative Dermatology*.

[27] Zhang Q, Lu Y, Proulx ST (2007). Increased lymphangiogenesis in joints of mice with inflammatory arthritis. *Arthritis Research and Therapy*.

[28] Ferrara N, Gerber HP, LeCouter J (2003). The biology of VEGF and its receptors. *Nature Medicine*.

[29] Hoeben A, Landuyt B, Highley MS, Wildiers H, van Oosterom AT, de Bruijn EA (2004). Vascular endothelial growth factor and angiogenesis. *Pharmacological Reviews*.

[30] Adams RH, Alitalo K (2007). Molecular regulation of angiogenesis and lymphangiogenesis. *Nature Reviews Molecular Cell Biology*.

[31] Fong GH, Rossant J, Gertsenstein M, Breitman ML (1995). Role of the Flt-1 receptor tyrosine kinase in regulating the assembly of vascular endothelium. *Nature*.

[32] Hiratsuka S, Minowa O, Kuno J, Noda T, Shibuya M (1998). Flt-1 lacking the tyrosine kinase domain is sufficient for normal development and angiogenesis in mice. *Proceedings of the National Academy of Sciences of the United States of America*.

[33] Joukov V, Sorsa T, Kumar V (1997). Proteolytic processing regulates receptor specificity and activity of VEGF-C. *The EMBO Journal*.

[34] Mäkinen T, Veikkola T, Mustjoki S (2001). Isolated lymphatic endothelial cells transduce growth, survival and migratory signals via the VEGF-C/D receptor VEGFR-3. *The EMBO Journal*.

[35] Kriehuber E, Breiteneder-Geleff S, Groeger M (2001). Isolation and characterization of dermal lymphatic and blood endothelial cells reveal stable and functionally specialized cell lineages. *Journal of Experimental Medicine*.

[36] Huggenberger R, Siddiqui SS, Brander D (2011). An important role of lymphatic vessel activation in limiting acute inflammation. *Blood*.

[37] Huggenberger R, Ullmann S, Proulx ST, Pytowski B, Alitalo K, Detmar M (2010). Stimulation of lymphangiogenesis via VEGFR-3 inhibits chronic skin inflammation. *Journal of Experimental Medicine*.

[38] Ristimäki A, Narko K, Enholm B, Joukov V, Alitalo K (1998). Proinflammatory cytokines regulate expression of the lymphatic endothelial mitogen vascular endothelial growth factor-C. *Journal of Biological Chemistry*.

[39] Baluk P, Yao LC, Feng J (2009). TNF-*α* drives remodeling of blood vessels and lymphatics in sustained airway inflammation in mice. *Journal of Clinical Investigation*.

[40] Cursiefen C, Chen L, Borges LP (2004). VEGF-A stimulates lymphangiogenesis and hemangiogenesis in inflammatory neovascularization via macrophage recruitment. *Journal of Clinical Investigation*.

[41] Chan LS (2008). Atopic dermatitis in 2008. *Current Directions in Autoimmunity*.

[42] Yano K, Oura H, Detmar M (2002). Targeted overexpression of the angiogenesis inhibitor thrombospondin-1 in the epidermis of transgenic mice prevents ultraviolet-B-induced angiogenesis and cutaneous photo-damage. *Journal of Investigative Dermatology*.

[43] Brown LF, Harrist TJ, Yeo KT (1995). Increased expression of vascular permeability factor (vascular endothelial growth factor) in bullous pemphigoid, dermatitis herpetiformis, and erythema multiforme. *Journal of Investigative Dermatology*.

[44] Detmar M (2000). The role of VEGF and thrombospondins in skin angiogenesis. *Journal of Dermatological Science*.

[45] Bhushan M, McLaughlin B, Weiss JB, Griffiths CEM (1999). Levels of endothelial cell stimulating angiogenesis factor and vascular endothelial growth factor are elevated in psoriasis. *British Journal of Dermatology*.

[46] Zenz R, Eferl R, Kenner L (2005). Psoriasis-like skin disease and arthritis caused by inducible epidermal deletion of Jun proteins. *Nature*.

[47] Raychaudhuri SP, Sanyal M, Raychaudhuri SK, Dutt S, Farber EM (2001). Severe combined immunodeficiency mouse-human skin chimeras: a unique animal model for the study of psoriasis and cutaneous inflammation. *British Journal of Dermatology*.

[48] Xia YP, Li B, Hylton D, Detmar M, Yancopoulos GD, Rudge JS (2003). Transgenic delivery of VEGF to mouse skin leads to an inflammatory condition resembling human psoriasis. *Blood*.

[49] Halin C, Fahrngruber H, Meingassner JG (2008). Inhibition of chronic and acute skin inflammation by treatment with a vascular endothelial growth factor receptor tyrosine kinase inhibitor. *American Journal of Pathology*.

[50] Schön MP (2008). Animal models of psoriasis: a critical appraisal. *Experimental Dermatology*.

[51] van der Fits L, Mourits S, Voerman JSA (2009). Imiquimod-induced psoriasis-like skin inflammation in mice is mediated via the IL-23/IL-17 axis. *Journal of Immunology*.

[52] Brewster CE, Howarth PH, Djukanovic R, Wilson J, Holgate ST, Roche WR (1990). Myofibroblasts and subepithelial fibrosis in bronchial asthma. *American Journal of Respiratory Cell and Molecular Biology*.

[53] Roche WR, Beasley R, Williams JH, Holgate ST (1989). Subepithelial fibrosis in the bronchi of asthmatics. *The Lancet*.

[54] Orsida BE, Li X, Hickey B, Thien F, Wilson JW, Walters EH (1999). Vascularity in asthmatic airways: relation to inhaled steroid dose. *Thorax*.

[55] Chetta A, Zanini A, Torre O, Olivieri D (2007). Vascular remodelling and angiogenesis in asthma: Morphological aspects and pharmacological modulation. *Inflammation and Allergy*.

[56] Hashimoto M, Tanaka H, Abe S (2005). Quantitative analysis of bronchial wall vascularity in the medium and small airways of patients with asthma and COPD. *Chest*.

[57] Dunill MS (1960). The pathology of asthma, with special reference to changes in the bronchial mucosa. *Journal of Clinical Pathology*.

[58] Asai K, Kanazawa H, Otani K, Shiraishi S, Hirata K, Yoshikawa J (2002). Imbalance between vascular endothelial growth factor and endostatin levels in induced sputum from asthmatic subjects. *Journal of Allergy and Clinical Immunology*.

[59] Hoshino M, Nakamura Y, Hamid QA (2001). Gene expression of vascular endothelial growth factor and its receptors and angiogenesis in bronchial asthma. *Journal of Allergy and Clinical Immunology*.

[60] Zanini A, Chetta A, Saetta M (2007). Chymase-positive mast cells play a role in the vascular component of airway remodeling inasthma. *Journal of Allergy and Clinical Immunology*.

[61] Gruber BL, Marchese MJ, Kew R (1995). Angiogenic factors stimulate mast-cell migration. *Blood*.

[62] Detmar M, Brown LF, Schön MP (1998). Increased microvascular density and enhanced leukocyte rolling and adhesion in the skin of VEGF transgenic mice. *Journal of Investigative Dermatology*.

[63] Barleon B, Sozzani S, Zhou D, Weich HA, Mantovani A, Marmé D (1996). Migration of human monocytes in response to vascular endothelial growth factor (VEGF) is mediated via the VEGF receptor flt-1. *Blood*.

[64] Feistritzer C, Kaneider NC, Sturn DH, Mosheimer BA, Kähler CM, Wiedermann CJ (2004). Expression and function of the vascular endothelial growth factor receptor FLT-1 in human eosinophils. *American Journal of Respiratory Cell and Molecular Biology*.

[65] Detoraki A, Staiano RI, Granata F (2009). Vascular endothelial growth factors synthesized by human lung mast cells exert angiogenic effects. *Journal of Allergy and Clinical Immunology*.

[66] McDonald DM (2001). Angiogenesis and remodeling of airway vasculature in chronic inflammation. *American Journal of Respiratory and Critical Care Medicine*.

[67] Chung KF, Rogers DF, Barnes PJ, Evans TW (1990). The role of increased airway microvascular permeability and plasma exudation in asthma. *European Respiratory Journal*.

[68] El-Chemaly S, Pacheco-Rodriguez G, Ikeda Y, Malide D, Moss J (2009). Lymphatics in idiopathic pulmonary fibrosis: new insights into an old disease. *Lymphatic Research and Biology*.

[69] Leibovich SJ, Polverini PJ, Shepard HM, Wiseman DM, Shively V, Nuseir N (1987). Macrophage-induced angiogenesis is mediated by tumour necrosis factor-*α*. *Nature*.

[70] Frater-Schroder M, Risau W, Hallmann R (1987). Tumor necrosis factor type *α*, a potent inhibitor of endothelial cell growth in vitro, is angiogenic in vivo. *Proceedings of the National Academy of Sciences of the United States of America*.

[71] Fajardo LF, Kwan HH, Kowalski J, Prionas SD, Allison AC (1992). Dual role of tumor necrosis factor-*α* in angiogenesis. *American Journal of Pathology*.

[72] Jagielska J, Kapopara PR, Salguero G (2012). Interleukin-1 assembles a proangiogenic signaling module consisting of caveolin-1, tumor necrosis factor receptor-associated factor 6, p38-mitogen-activated protein kinase (MAPK), and MAPK-activated protein kinase 2 in endothelial cells. *Arteriosclerosis, Thrombosis and Vascular Biology*.

[73] BenEzra D, Hemo I, Maftzir G (1990). In vivo angiogenic activity of interleukins. *Archives of Ophthalmology*.

[74] Cozzolino F, Torcia M, Aldinucci D (1990). Interleukin 1 is an autocrine regulator of human endothelial cell growth. *Proceedings of the National Academy of Sciences of the United States of America*.

[75] Koch AE, Polverini PJ, Kunkel SL (1992). Interleukin-8 as a macrophage-derived mediator of angiogenesis. *Science*.

[76] Addison CL, Daniel TO, Burdick MD (2000). The CXC chemokine receptor 2, CXCR2, is the putative receptor for ELR+ CXC chemokine-induced angiogenic activity. *Journal of Immunology*.

[77] Angiolillo AL, Kanegane H, Sgadari C, Reaman GH, Tosato G (1997). Interleukin-15 promotes angiogenesis in vivo. *Biochemical and Biophysical Research Communications*.

[78] Numasaki M, Fukushi JI, Ono M (2003). Interleukin-17 promotes angiogenesis and tumor growth. *Blood*.

[79] Park CC, Morel JCM, Amin MA, Connors MA, Harlow LA, Koch AE (2001). Evidence of IL-18 as a novel angiogenic mediator. *Journal of Immunology*.

[80] Amin MA, Rabquer BJ, Mansfield PJ (2010). Interleukin 18 induces angiogenesis in vitro and in vivo via Src and Jnk kinases. *Annals of the Rheumatic Diseases*.

[81] Coughlin CM, Salhany KE, Wysocka M (1998). Interleukin-12, and interleukin-18 synergistically induce murine tumor regression which involves inhibition of angiogenesis. *Journal of Clinical Investigation*.

[82] Cao R, Farnebo J, Kurimoto M, Cao Y (1999). Interleukin-18 acts as an angiogenesis and tumor suppressor. *The FASEB Journal*.

[83] Strieter RM, Polverini PJ, Kunkel SL (1995). The functional role of the ELR motif in CXC chemokine-mediated angiogenesis. *Journal of Biological Chemistry*.

[84] Keeley EC, Mehrad B, Strieter RM (2008). Chemokines as mediators of neovascularization. *Arteriosclerosis, Thrombosis, and Vascular Biology*.

[85] Fan Y, Ye J, Shen F (2008). Interleukin-6 stimulates circulating blood-derived endothelial progenitor cell angiogenesis in vitro. *Journal of Cerebral Blood Flow and Metabolism*.

[86] Volin MV, Woods JM, Amin MA, Connors MA, Harlow LA, Koch AE (2001). Fractalkine: a novel angiogenic chemokine in rheumatoid arthritis. *American Journal of Pathology*.

[87] Salcedo R, Wasserman K, Young HA (1999). Vascular endothelial growth factor and basic fibroblast growth factor induce expression of CXCR4 on human endothelial cells. In vivo neovascularization induced by stromal-derived factor-1*α*. *American Journal of Pathology*.

[88] Kryczek I, Frydman N, Gaudin F (2005). The chemokine SDF-1/CXCL12 contributes to T lymphocyte recruitment in human pre-ovulatory follicles and coordinates with lymphocytes to increase granulosa cell survival and embryo quality. *American Journal of Reproductive Immunology*.

[89] Salcedo R, Ponce ML, Young HA (2000). Human endothelial cells express CCR2 and respond to MCP-1: direct role of MCP-1 in angiogenesis and tumor progression. *Blood*.

[90] Ebnet K, Vestweber D (1999). Molecular mechanisms that control leukocyte extravasation: the selectins and the chemokines. *Histochemistry and Cell Biology*.

